# A study of the *TNF/LTA/LTB *locus and susceptibility to severe malaria in highland papuan children and adults

**DOI:** 10.1186/1475-2875-9-302

**Published:** 2010-10-29

**Authors:** Louise M Randall, Enny Kenangalem, Daniel A Lampah, Emiliana Tjitra, Esther D Mwaikambo, Tjandra Handojo, Kim A Piera, Zhen Z Zhao, Fabian de Labastida Rivera, Yonghong Zhou, Karli M McSweeney, Lien Le, Fiona H Amante, Ashraful Haque, Amanda C Stanley, Tonia Woodberry, Ervi Salwati, Donald L Granger, Maurine R Hobbs, Ric N Price, J Brice Weinberg, Grant W Montgomery, Nicholas M Anstey, Christian R Engwerda

**Affiliations:** 1 Queensland Institute of Medical Research and Australian Centre for Vaccine Development, 300 Herston Road, Herston, QLD 4006, Australia; 2 The University of Queensland, School of Population Health, Herston Road, Herston, QLD 4006, Australia; 3 National Institute of Health Research and Development-Menzies School of Health Research Malaria Research Programme, and District Ministry of Health, Timika, Papua, Indonesia; 4 National Institute of Health Research and Development, Jakarta, Indonesia; 5 Department of Paediatrics, Herbert Kairuki Memorial University, Dar es Salaam, Tanzania; 6 International Health Division, Menzies School of Health Research and Charles Darwin University, Darwin, NT, Australia; 7 Divisions of Infectious Diseases and Endocrinology, University of Utah and VA Medical Centers, Utah, USA; 8 Centre for Vaccinology & Tropical Medicine, Nuffield Department of Clinical Medicine, Churchill Hospital, Oxford, UK; 9 Division of Medicine, Royal Darwin Hospital, Darwin, NT, Australia; 10 Division of Hematology-Oncology, Duke and VA Medical Centers, Durham, NC, USA

## Abstract

**Background:**

Severe malaria (SM) syndromes caused by *Plasmodium falciparum *infection result in major morbidity and mortality each year. However, only a fraction of *P. falciparum *infections develop into SM, implicating host genetic factors as important determinants of disease outcome. Previous studies indicate that tumour necrosis factor (TNF) and lymphotoxin alpha (LTα) may be important for the development of cerebral malaria (CM) and other SM syndromes.

**Methods:**

An extensive analysis was conducted of single nucleotide polymorphisms (SNPs) in the *TNF, LTA *and *LTB *genes in highland Papuan children and adults, a population historically unexposed to malaria that has migrated to a malaria endemic region. Generated *P*-values for SNPs spanning the *LTA/TNF/LTB *locus were corrected for multiple testing of all the SNPs and haplotype blocks within the region tested through 10,000 permutations. A global P-value of < 0.05 was considered statistically significant.

**Results:**

No associations between SNPs in the *TNF/LTA/LTB *locus and susceptibility to SM in highland Papuan children and adults were found.

**Conclusions:**

These results support the notion that unique selective pressure on the *TNF/LTA/LTB *locus in different populations has influenced the contribution of the gene products from this region to SM susceptibility.

## Background

Severe malaria (SM) caused by *Plasmodium falciparum *results in more than a million deaths each year [1,2]. It is a collection of syndromes that includes cerebral malaria (CM), severe malaria anaemia (SMA), acute respiratory distress syndrome (ARDS), hyperparasitaemia, hypoglycaemia, black water fever, metabolic acidosis, jaundice and renal failure [1]. The reasons why some individuals develop severe complications of malaria, whereas others do not, are still unclear. However, the virulence of the parasite strain causing malaria, as well as the age and genetic background of the infected individual are likely to influence disease outcome.

*Plasmodium falciparum *and humans have had intense interactions for at least 6,000 years and it is thought that malaria has imposed a large selective pressure on the human genome [3,4]. Evidence from both an experimental cerebral malaria (ECM) model [5], as well as from SM patients [6-8], have identified TNF as an important pro-inflammatory cytokine for the control of infection, and also a strong association with the development of pathology. Single nucleotide polymorphisms (SNPs) within the gene encoding TNF (*TNF*) have been associated with severe outcomes following *Plasmodium *infection in a number of populations in Africa, Asia and the Pacific region [9-13]. In particular, a SNP in the promoter region of *TNF *at 308 nucleotide base pairs upstream of the transcription start site (-308/376*TNF*) has been reported to confer a greater risk of severe neurological sequelae or death due to CM in the Gambia [14]. This variant was found to be a stronger transcriptional activator than the common allele in some *in vitro *studies [15], but not others [16]. More recent studies examining the MHC class III region, which includes *TNF *as well as the genes encoding the closely related LTα (*LTA*) and LTβ (*LTB*), suggest that positive associations between disease and *TNF *alleles could in fact be due to real disease alleles in neighbouring genes [17]. One such study observed that when specific SNPs were present in TNF, in combination with specific SNPs in *LTA*, *LTA *transcription rather than *TNF *transcription was modulated by changing the way RNA polymerase specifically bound to the *LTA *promoter [18]. Importantly, LTα, not TNF, has been shown to be a critical factor in the development of experimental cerebral malaria in C57BL/6 mice [19], identifying *LTA *along with *TNF *as a candidate susceptibility gene.

Recently, a genome wide association study involving 3 African populations from The Gambia, Kenya and Malawi, investigated potential associations between SM and 8 SNPs spanning the *LTA/TNF *locus [20]. An association was found between *TNF*-238A allele and SM in samples from The Gambia only, and not for any other SNP tested in any of the populations examined [20]. These findings suggested different selective pressures in the *LTA/TNF *locus in different populations, as well as highlighting the need for more-detailed mapping of polymorphisms across this locus to identify causal SNPs associated with SM susceptibility [20]. Here, an extensive analysis was conducted of 35 SNP's found in the *LTA/TNF/LTB *gene locus in adults and children of highland Papuan origin, a population not historically exposed to malaria transmission prior to the 1970 s, but who during the study period had been exposed to malaria following migration to the lowland region of Papua.

## Methods

### Study participants and sample preparation

Characteristics of the study participants upon admission to hospital and controls, including population structure, have been described in Table 1 and the methods section of a recent publication [21]. No corrections were required. Highland Papuan patients with SM and asymptomatic malaria-exposed controls were recruited in a case-control study in Timika, a lowland region of Papua, Indonesia. Genotypes significantly associated with SM in highland Papuans, were also examined in a Tanzanian case-control study comprising children with CM enrolled in Dar es Salaam using WHO criteria as previously described [22] and asymptomatic malaria-exposed, healthy control children from Mikocheni Primary school in the Kinondoni Municipality of the Dar es Salaam region. Written informed consent was obtained from all study subjects or their next of kin, parent or guardian. Studies were approved by the Ethics Committees of the National Institute of Health Research and Development (Ministry of Health, Jakarta, Indonesia), Menzies School of Health Research (Darwin, Australia), Queensland Institute of Medical Research (Brisbane, Australia), Muhimbili University of Health Sciences (Dar es Salaam, Tanzania), National Institute for Medical Research (Dar es Salaam, Tanzania), University of Utah Medical Center (Salt Lake City, USA) and Duke University Medical Center (Durham, USA).

**Table 1:** The SNP set used to investigate the *LTA/TNF/LTB *locus.

Map Number	db SNP rs	SNP Location	Gene	Role	Polymorphic
1	rs2857602	chr6:31641357	LTA	Promoter	Yes
2	rs2844486	chr6:31641849	LTA	Promoter	No
3	rs3131637	chr6:31643053	LTA	Promoter	Yes
4	rs2844484	chr6:31644203	LTA	Promoter	Yes
5	rs2844483	chr6:31644775	LTA	Promoter	Yes
6	rs2009658	chr6:31646223	LTA	Promoter	Yes
7	rs4647191	chr6:31646617	LTA	Promoter	No
8	rs2844482	chr6:31647746	LTA	Promoter	Yes
9	rs2071590	chr6:31647747	LTA	Promoter	Yes
10	rs1800683	chr6:31648050	LTA	Promoter	Yes
11	rs2239704	chr6:31648120	LTA	Exon	Yes
12	rs909253	chr6:31648292	LTA	Intron (boundary)	Yes
13	rs2857713	chr6:31648535	LTA	Coding exon	Yes
14	rs3093543	chr6:31648736	LTA	Coding exon	No
15	rs1041981	chr6:31648763	LTA	Coding exon	Yes
16	rs1799964	chr6:31650287	LTA	3' UTR	Yes
17	rs1799724	chr6:31650461	LTA	3' UTR	Yes
18	rs1800750	chr6:31650942	TNF	Promoter	No
19	rs1800629	chr6:31651010	TNF	Promoter	No
20	rs361525	chr6:31651080	TNF	Promoter	No
21	rs3179060	chr6:31651651	TNF	Coding exon	No
22	rs3093661	chr6:31651737	TNF	Intron (boundary)	No
23	rs1800610	chr6:31651806	TNF	Intron	Yes
24	rs3093662	chr6:31652168	TNF	Intron	No
25	rs4645843	chr6:31652541	TNF	Coding exon	No
26	rs1800620	chr6:31652570	TNF	Intron (boundary)	No
27	rs3093664	chr6:31652621	TNF	Intron (boundary)	No
28	rs1800618	chr6:31652897	TNF	Coding exon	No
29	rs11574936	chr6:31653172	TNF	Coding exon	No
30	rs3093668	chr6:31654474	TNF	3' UTR	No
31	rs3091257	chr6:31654829	TNF	3' UTR	Yes
32	rs769178	chr6:31655493	LTB	3' UTR	Yes
33	rs769177	chr6:31655590	LTB	3' UTR	Yes
34	rs2229699	chr6:31656835	LTB	Exon	No
35	rs1052248	chr6:31664560	LST1^a^	Exon	Yes

^a^LST1, leukocyte specific transcript 1 (also the 5' UTR of *LTB*).

### Sequencing the LTA gene

The gene encoding LTα, spanning a region of 5323 base pairs (bp) was sequenced in a subset of samples to check for the presence of novel SNPs. The locus was amplified in sections of approximately 500 bp by PCR and sequenced. Sequenced products were aligned against a published human *LTA *sequence [23]. Identified SNPs were also checked on the NCBI website [24] for previous publication.

### SNP selection

*TNF/LTA/LTB *SNPs were included in this study based on reported functional changes (i.e. transcriptional or protein level) or on previous associations with malaria, other infectious diseases, ischaemic stroke, cerebral infarction, atherosclerosis or inflammation [9-12,14,18,25-43]. SNPs in the *TNF/LTA/LTB *locus observed to be present in ethnic groups from Sulawesi, an island in the Indonesian archipelago [44], were also included. The final set of polymorphisms included as many functional and disease-associated SNPs as possible following primer design and testing. Tagging SNPs were selected based on reported frequencies in other populations. This SNP set has been described elsewhere [45]. All SNP sequences were obtained from the Chip Bioinformatics database [46] and verified in NCBI.

### Genotyping

Assays were designed for thirty-five SNPs across the *TNF/LTA/LTB *region in a multiplex using the Sequenom MassARRAY Assay Design software (version 3.0). SNPs were typed using iPLEX™ chemistry and analyzed using a Sequenom MassARRAY Compact Mass Spectrometer (Sequenom Inc, San Diego, CA, USA). The 2.5 ml PCR reactions were performed in standard 384-well plates using 10 ng genomic DNA, 0.5 unit of Taq polymerase (HotStarTaq, Qiagen), 500 mmol of each dNTP, and 100 nmol of each PCR primer. Standard PCR thermal cycling conditions and post-PCR extension reactions were carried out as described previously [45]. The iPLEX reaction products were desalted and spotted on a SpectroChip (Sequenom). Data were processed and analysed by MassARRAY Workstation (version 3.4) software (Sequenom). Single SNP genotyping specific for rs2071590 and rs1052248 was performed using TaqMan SNP Genotyping Assays according to the manufacturer's instructions (Applied Biosystems, Foster City, CA) by using the allelic discrimination on a Corbett RG-6000 (Corbett Life Sciences, Sydney, NSW, Australia) or an AB7900 machine (Applied Biosystems). Previously characterized genotypes for these SNPs were included as positive controls and run alongside samples with unknown genotype.

### Statistical analyses

Based on our sample size (380 cases and 356 controls), 80% power was available to detect a disease allele with a relative risk of 1.5 at a disease frequency of 0.25. Haploview version 3.32 (Whitehead Institute for Biomedical Research, USA; [47]) was used to perform all statistical tests relating to this SNP analysis [48]. Genotype frequencies of all SNPs were tested for departures from Hardy-Weinberg equilibrium in both cases and controls separately. In the Papuan study, 16 SNPs were found to be non-polymorphic (Table 1). Haplotype frequencies and linkage disequilibrium (LD) tests were also determined by Haploview version 3.32 [48] using the default method of Gabriel [49]. The association between single markers and haplotype blocks was performed by the Haploview programme. Generated *P*-values for SNPs spanning the *LTA/TNF/LTB *locus were corrected for multiple testing of all the SNPs and haplotype blocks within the region tested through 10,000 permutations. A global P-value of < 0.05 was considered statistically significant.

## Results

### LTA/TNF locus is not associated with severe malaria

**Figure 1:**
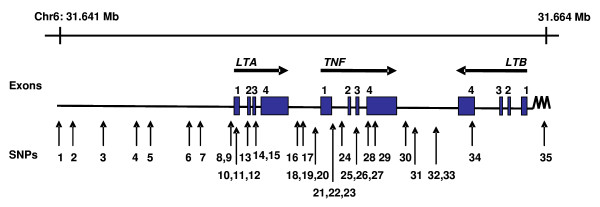
**Chromosomal location of SNPs studied in the *LTA/TNF/LTB *locus**. 35 SNPs that cover the *LTA/TNF/LTB *locus on chromosome 6 were analysed in highland Papuans. SNPs selected for study were based on reported functional changes, previous associations with malaria, and other infectious diseases or inflammatory conditions. Certain tagged SNPs were included based on reported frequencies in other populations.

**Figure 2:**
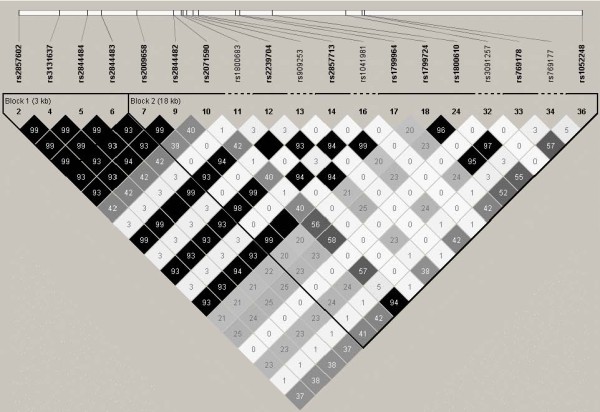
**Linkage disequilibrium plot of *LTA/TNF/LTB *SNP set in all severe malaria patients**. The LD plot shading scheme illustrates the estimated LD between the SNPs, and the LD value is displayed within the box. Dark grey regions signify strong LD (1.0), whereas light grey and white regions depict low LD (< 1.0).

In total, 380 SM cases (262 adults and 118 children) and 356 control individuals (305 adults and 51 children) were included in the genotyping study [21]. A subset of the samples was randomly chosen for sequencing of a 5,323 bp region spanning *LTA*, and this revealed no novel SNPs in the study population, and that all SNPs detected in the sequenced *LTA *had previously been reported and, where possible, were included in the SNP set (Table 1). The SNP set consisted of 35 SNPs that spanned the *LTA/TNF/LTB *locus on chromosome 6 (Figure 1 and Table 1). The majority of the samples were successfully genotyped for each of the SNPs with an average coverage of greater than 99%. Of these, 16 SNPs were found to be non-polymorphic (Table 1). The minor allele frequencies of the remaining SNPs spanning the *LTA/TNF/LTB *locus ranged from 0.001 to 0.44 in control subjects and from 0.001 to 0.49 in patients with SM. Two SNPs, rs2071590 and rs1052248, situated in the *LTA *and *LTB *promoter regions, respectively, showed some differences in allele frequencies between control subjects and SM patients (*P *= 0.034 and *P *= 0.007, respectively). These differences were not observed once corrections were made for multiple testing (global *P *> 0.05; Table 2). Analysis of haplotypes did not reveal any association between haplotype blocks and susceptibility to SM (Figure 2 and Table 3).

**Table 2:** Association analysis of SNPs across the *LTA/TNF/LTB *gene locus genotyped in severe malaria cases and controls.

dbSNP rs	Position	Gene	Role	Alleles	MAF^a^		OR^b^ (CI)^c^	Association^d^ χ^2^	*P-*value
								
					Controls	SM			
rs2857602	chr6:31641357	LTA	Promoter	G > A	0.27	0.27	1.02 (0.81-1.28)	0.02	1.000
rs3131637	chr6:31643053	LTA	Promoter	A > T	0.27	0.27	1.02 (0.81-1.28)	0.02	1.000
rs2844484	chr6:31644203	LTA	Promoter	A > G	0.27	0.27	1.03 (0.81-1.29)	0.05	1.000
rs2844483	chr6:31644775	LTA	Promoter	T > G	0.27	0.27	1.02 (0.81-1.28)	0.02	1.000
rs2009658	chr6:31646223	LTA	Promoter	C > G	0.25	0.26	1.05 (0.83-1.33)	0.14	1.000
rs2844482	chr6:31647746	LTA	Promoter	C > T	0.25	0.26	1.07 (0.85-1.35)	0.30	1.000
rs2071590	chr6:31647747	LTA	Promoter	A > G	0.44	0.49	1.25 (1.02-1.54)	4.52	0.366
rs1800683	chr6:31648050	LTA	Promoter	G > A	0.02	0.01	1.98 (0.72-5.35)	1.82	0.979
rs2239704	chr6:31648120	LTA	Exon	A > C	0.27	0.27	1.02 (0.81-1.29)	0.03	1.000
rs909253	chr6:31648292	LTA	Intron (boundary)	A > G	0.02	0.01	1.98 (0.73-5.36)	1.84	0.978
rs2857713	chr6:31648535	LTA	Coding exon	T > C	0.25	0.26	1.06 (0.84-1.34)	0.23	1.000
rs1041981	chr6:31648763	LTA	Coding exon	C > A	0.02	0.01	2.15 (0.80-5.77)	2.44	0.814
rs1799964	chr6:31650287	LTA	3' UTR	T > C	0.25	0.27	1.07 (0.84-1.35)	0.30	1.000
rs1799724	chr6:31650461	LTA	3' UTR	C > T	0.43	0.39	1.19 (0.97-1.47)	2.76	0.755
rs1800610	chr6:31651806	TNF	Intron	G > A	0.42	0.39	1.13 (0.92 - 1.40)	1.37	0.992
rs3091257	chr6:31654829	TNF	3' UTR	C > A	< 0.01	< 0.01	1.07 (0.07 - 17.11)	< 0.01	1.000
rs769178	chr6:31655493	LTB	3' UTR	C > A	0.43	0.39	1.20 (0.97 - 1.49)	2.98	0.682
rs769177	chr6:31655590	LTB	3' UTR	G > A	0.04	0.05	1.11 (0.67 - 1.84)	0.15	1.000
rs1052248	chr6:31664560	LST1	Exon	A > T	0.42	0.49	1.33 (1.08 -1.63)	7.22	0.098

^a^Minor allele frequency.

^b^Odds ratio.

^c^95% confidence interval.

^d^Association χ^2 ^with severe malaria.

**Table 3:** Common haplotype blocks and their association with severe malaria

Haplotype	Frequency (control)	c^2^	*P-value*
Block 1:			
GAAT	0.73	0.01	1.000
ATGG	0.27	0.01	1.000
Block 2:			
CCAATTTAAA	0.42	1.38	0.992
GTGCCCCGCT	0.24	0.56	1.000
CCGATTCGCT	0.17	6.63	0.133
CCAATTCGCA	0.14	1.64	0.984

Next, the *TNF/LTA/LTB *locus was examined to establish whether it was associated with susceptibility to CM in either adults or children. Overall, there were no differences in allele frequencies between control subjects and CM patients (Table 4). Furthermore, neither age nor susceptibility to CM was associated with the *TNF/LTA/LTB *locus for either single SNPs or haplotype blocks (Tables 3 and 4; Figure 2). Given the association between the two SNPs situated in the *LTA *and *LTB *promoter regions and SM prior to correcting for multiple testing described above, the association of rs2071590 and rs1052248 in patients originating from and resident in a malaria-endemic region (245 healthy children (HC) and 77 children with CM from Dar es Salaam, Tanzania) was also examined, but no association between these SNPs and CM was found. Taken together, there was no evidence for the *TNF/LTA/LTB *locus contributing to SM or CM in highland Papuans historically not exposed to malaria.

**Table 4:** Lack of association between *LTA/TNF/LTB *SNPs and severe malaria subgroups when compared with adult and childhood controls in the Papuan population.

dbSNP rs	All CM (106)^a^		Adult SM (262)		Adult CM (85)		Childhood SM(118)		Childhood CM(21)	
	MAF^b^	*P*-value	MAF	*P*-value	MAF	*P*-value	MAF	*P*-value	MAF	*P*-value
rs2857602	0.28	1.000	0.27	1.000	0.27	1.000	0.28	0.999	0.26	1.000
rs3131637	0.28	1.000	0.27	1.000	0.27	1.000	0.28	0.999	0.26	1.000
rs2844484	0.28	1.000	0.27	1.000	0.27	1.000	0.28	0.999	0.26	1.000
rs2844483	0.28	1.000	0.27	1.000	0.27	1.000	0.28	0.999	0.26	1.000
rs2009658	0.25	1.000	0.26	1.000	0.26	1.000	0.26	0.951	0.22	0.999
rs2844482	0.27	1.000	0.26	1.000	0.26	1.000	0.26	0.957	0.22	0.999
rs2071590	0.51	0.841	0.46	0.895	0.45	0.963	0.47	0.631	0.44	0.950
rs1800683	0.01	1.000	0.01	1.000	0.01	1.000	0.02	0.488	0.04	1.000
rs2239704	0.28	1.000	0.27	1.000	0.27	1.000	0.28	0.999	0.26	1.000
rs909253	0.01	1.000	0.01	1.000	0.01	1.000	0.02	0.488	0.04	1.000
rs2857713	0.27	1.000	0.26	1.000	0.26	1.000	0.26	0.983	0.23	1.000
rs1041981	0.01	1.000	0.01	0.999	0.01	1.000	0.02	0.488	0.04	1.000
rs1799964	0.27	1.000	0.26	1.000	0.26	1.000	0.26	0.951	0.22	0.999
rs1799724	0.36	0.597	0.41	0.992	0.41	0.869	0.41	0.744	0.45	0.992
rs1800610	0.36	0.902	0.40	1.000	0.40	0.945	0.41	0.828	0.44	0.996
rs3091257	-	-	-	-	-	-	-	-	-	-
rs769178	0.36	0.581	0.41	0.992	0.41	0.851	0.41	0.83	0.44	0.996
rs769177	0.06	0.983	0.04	1.000	0.04	1.000	0.05	1.00	0.06	0.995
rs1052248	0.50	0.490	0.45	0.543	0.44	0.878	0.46	0.26	0.40	0.884

^a^Number in each group.

^b^MAF - minor allele frequency in cases.

## Discussion

Both genetic and serological data indicate roles for TNF and LTα in the pathogenesis of SM [6-14,50,51]. However, there was a lack of association between SNPs in the *TNF/LTA/LTB *and SM in a highland Papuan population. This lack of association remained following subsequent testing in disease subsets and in both children and adults. *TNF *polymorphisms have been associated with malaria transmission and SM, primarily in populations originating and living in malaria endemic areas [9-14]. A small number of studies have investigated the relationship between two *LTA *SNPs and malaria. *LTA *C+80A (rs2239704), a SNP that allows specific binding of the transcriptional repressor ABF-1 and, therefore, considered to be a low LTα-producing allele, has been associated with lower *P. falciparum *parasitaemia in malaria-endemic Burkina Faso but was not associated with SM in a case-control study in The Gambia [40,51,52]. *LTA *A+252G (rs909253) has been reported to influence LTα production [53], but this SNP was not associated with SM in Sri Lanka [9]. More recently, rs2239704 and rs909253 were reported to not be associated with SM in a study of SM patients from The Gambia, Kenya and Malawi [20]. Both rs2239704 and rs909253 were included in the present study but not found to be associated with SM. A recent genome wide association study found that only rs2516486 at *TNF *was weakly associated with malaria severity, however the authors caution that the candidate SNPs examined in this study were poorly tagged by the 500 K array used [54].

*LTA *polymorphisms were of particular interest. In highland Papuans (a population without historical exposure to malaria), the minor allele for rs2239704 (Table 3) is the opposite allele to that observed in a study population from malaria-endemic Burkina Faso [51], suggesting that different pressures may have selected for different alleles in the two populations. Despite two SNPs situated in the *LTA *and *LTB *promoter regions (rs2071590 and rs1052248, respectively), having differences in allele frequencies between control subjects and SM patients, this was not significant after correction for multiple testing, nor was there a difference in the Tanzanian population studied. Thus, our data provide no evidence for an involvement of the *LTA/TNF/LTB *locus in SM susceptibility in highland Papuans, and suggest that if the genes encoded by this locus are involved in SM pathogenesis then molecules that regulate the production and/or bioavailablity may influence disease outcome. Interestingly, an association between susceptibility to SM and a SNP in the LTα-related gene encoding galectin-2 (*LGALS2*) was recently identified. The particular SNP in *LGALS2 *is thought to regulate the trafficking of LTα out of cells [55]. Strikingly, the association between *LGALS2 *and SM was found to be present in highland Papuan children, but not adults [21]. A further consideration in future studies should be the effects of other genes with important immunological functions in the region surrounding the TNF locus that could contribute to the development of severe malaria. The potential importance of such genes was highlighted in a recent study showing that SNPs in HLA-B associated transcript 2 (*BAT2*) in the MHC III region were associated with severe malaria susceptibility, while *TNF *and *LTA *SNPs were not [52].

## Conclusion

It is clear that large, highly powered studies will be necessary to identify causal variants in genes that contribute to SM disease outcome. However, as discussed above, it is likely that different populations have had different selective pressures placed upon them, resulting in a number of susceptibility alleles across different populations. Hence, deep sequencing studies in the *TNF/LTA/LTB *locus of different populations may be warranted and required to identify causal variants in these genes responsible for susceptibility to SM syndromes.

## List of abbreviations

SM: severe malaria; TNF: tumour necrosis factor; LT: lymphotoxin; CM: cerebral malaria; SNP: single nucleotide polymorphism; SMA: severe malaria anaemia; ARDS: acute respiratory distress syndrome; bp: base pair; LD: linkage disequilibrium.

## Competing interests

The authors declare that they have no competing interests.

## Authors' contributions

LMR performed experiments, analysed data and drafted the manuscript, EK, DAL, ET, EDM, TH, KP, ZZZ, FLR, YZ, KMS, LL, FHA, AH, ACS, TW and ES collected and processed samples, and performed experiments, DLG, MRH, RNP, JBW, GWM, NMA and CRE designed experiments analysed data and wrote the manuscript. All authors read and approved the final manuscript.
